# Improving naturalistic neuroscience with patient engagement strategies

**DOI:** 10.3389/fnhum.2023.1325154

**Published:** 2024-01-08

**Authors:** Lucia K. Feldmann, Juliet Roudini, Andrea A. Kühn, Jeroen G. V. Habets

**Affiliations:** ^1^Movement Disorder and Neuromodulation Unit, Department of Neurology, Charité – Universitätsmedizin Berlin, Berlin, Germany; ^2^QUEST Center for Responsible Research, Berlin Institute of Health at Charité, Berlin, Germany; ^3^Patient and Stakeholder Engagement, Cluster of Excellence, NeuroCure, Berlin, Germany; ^4^Berlin School of Mind and Brain, Charité University Medicine, Berlin, Germany; ^5^NeuroCure Clinical Research Center, Charité University Medicine, Berlin, Germany; ^6^DZNE, German Center for Neurodegenerative Diseases, Berlin, Germany

**Keywords:** deep brain stimulation, patient engagement, Parkinson’s disease, neurophysiology, home monitoring, naturalistic monitoring

## Abstract

**Introduction:**

The clinical implementation of chronic electrophysiology-driven adaptive deep brain stimulation (DBS) algorithms in movement disorders requires reliable representation of motor and non-motor symptoms in electrophysiological biomarkers, throughout normal life (naturalistic). To achieve this, there is the need for high-resolution and -quality chronic objective and subjective symptom monitoring in parallel to biomarker recordings. To realize these recordings, an active participation and engagement of the investigated patients is necessary. To date, there has been little research into patient engagement strategies for DBS patients or chronic electrophysiological recordings.

**Concepts and results:**

We here present our concept and the first results of a patient engagement strategy for a chronic DBS study. After discussing the current state of literature, we present objectives, methodology and consequences of the patient engagement regarding study design, data acquisition, and study infrastructure. Nine patients with Parkinson’s disease and their caregivers participated in the meeting, and their input led to changes to our study design. Especially, the patient input helped us designing study-set-up meetings and support structures.

**Conclusion:**

We believe that patient engagement increases compliance and study motivation through scientific empowerment of patients. While considering patient opinion on sensors or questionnaire questions may lead to more precise and reliable data acquisition, there was also a high demand for study support and engagement structures. Hence, we recommend the implementation of patient engagement in planning of chronic studies with complex designs, long recording durations or high demand for individual active study participation.

## Introduction

Deep brain stimulation (DBS) is an effective therapy for movement disorders. Besides its clinical relevance for symptom alleviation, DBS enables electrophysiological recordings from pathophysiologically relevant deep brain structures. Especially for Parkinson’s disease (PD), DBS led to knowledge about symptom-specific electrophysiological signatures (Brown and Thompson, 2001; Kühn et al., 2006; Tinkhauser et al., 2017; Feldmann et al., 2022) applied as a feedback signal for personalized adaptive stimulation (aDBS) (Little et al., 2013; Arlotti et al., 2018; Velisar et al., 2019). First studies with clinical application of aDBS are under way (Caffi et al., 2023; Herrington et al., 2023). However, most physiomarker research to date has a limited ecological validity due to its highly controlled lab conditions and short recording durations. For ecologically valid, real-life (naturalistic) physiomarker data, additional data on patient wellbeing and symptom severity is necessary for data interpretation. For successful data collection, we implemented patient engagement in our study design. Here, we describe our perspective and experiences.

**FIGURE 1:**
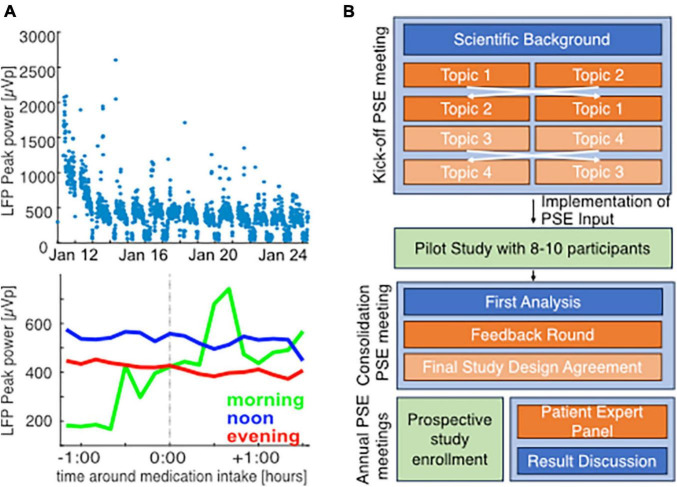
Challenges of chronic monitoring and concept for patient engagement in studies on naturalistic neuroscience in PD patients: single example for illustration of challenges in chronic biomarker acquisition in a 56-year old male PD patient with subthalamic DBS and peak biomarker recording at 22 Hz (right hemisphere). Data acquisition and analysis was performed as previously described [van Rheede et al., 2022; Feldmann et al., 2023, approved by the local ethics committee (EA2/256/60)] in parallel with clinical records at a post-operative rehabilitation. At a low-resolution of 3-days means, beta band suppression from the beginning toward the end of the stay reveals that clinical improvement through therapy optimization is also reflected in biomarker levels (start of rehab: 758.9 ± 400.7, end of rehab 273.96 ± 160.3, mean ± standard deviation), (**A**, top). However, higher resolution analysis of 10 min-mean beta band activity around medication intake based on clinical notes (mean for morning: 9, noon: 32, evening: 21 time points) does not lead to conclusive results and may be influenced by circadian rhythms (**A**, bottom). This demands better quality symptomatic documentation, that requires a high level of patient commitment. Therefore, we developed a patient engagement strategy **(B)**. Orange panels indicate active patient involvement throughout the study, blue panels indicate the scientific information discussed with patients, and green panels indicate the data acquisition phases.

Parkinson’s disease (PD) is a systemic disease characterized by heterogeneous motor and non-motor symptomatology that may require diverse aDBS-physiomarkers. Various factors besides solely symptom fluctuation will modulate these symptom-specific physiomarkers during normal life. Reliable symptomatic and electrophysiological monitoring for PD and DBS may not only answer urgent scientific questions, but also increase the accessibility of the emerging global PD patient care (Bloem et al., 2021), especially in medically underserved areas, e.g., by online therapy optimization in remote areas (Klucken et al., 2018; Bloem et al., 2020; Virmani et al., 2022). Therefore, a thorough understanding of the naturalistic variability of PD symptoms and its physiomarkers on an intra-day and inter-day level is important to establish aDBS paradigms. In our first experiences with naturalistic chronic subthalamic recordings, we demonstrated how factors like circadian periodicity and movement modulate the currently proposed PD-physiomarker (Figure 1A, top) (van Rheede et al., 2022). However, higher resolution analyses focusing on i.e., single medication-intake moments appeared to be less conclusive and would likely benefit from additional detailed symptomatic and contextual information (Figure 1A, bottom).

To elucidate this, we can collect symptomatic data in parallel to the chronic neural recordings in an active or passive manner. Active data requires an active patient involvement, such as completing a patient reported outcome (PRO) (Habets et al., 2020; Weizenbaum et al., 2022) or participating in a motor task, i.e., tapping task, (Zhan et al., 2018; Lipsmeier et al., 2022). Passive data collection is done unobtrusively via i.e., inertial measurement units (IMUs) containing kinematic sensors i.e., heart rate, or geolocation (Cornet and Holden, 2018; De Calheiros Velozo et al., 2022). For valid and meaningful data analysis, it is of key importance that the parallel data captures the relevant symptomatology reliably, contains essential contextual information on e.g., medication intakes, and above all, performs data acquisition as continuously as possible. For this, the primary necessity is study participant compliance. Hence, we believe we can only achieve high quality, active patient participation by involving those people during the study planning that are at the center of our research: the patients themselves.

## Current literature

The inclusion of patients in the definition of research-agendas and study designs can improve studies’ feasibility, cost-effectiveness, and validity (Schipper et al., 2014; Roudini et al., 2023). Sporadically, investigators pioneered patient engagement in PD studies and reported about this endeavor (Meinders et al., 2022; Evers et al., 2023). Meinders and colleagues recently published general advice and experiences with patient engagement in PD studies, but specific reports on patient engagement for naturalistic monitoring studies with DBS patients lack, to our best knowledge.

So far, only a few studies reported the implementation of patient engagement methods to improve home monitoring studies with PD patients. An international, multi-center study included PD patients and healthcare professionals to define the most important symptoms and concepts of activities of daily life (ADL) to monitor during daily life (Ferreira et al., 2015; Serrano et al., 2015). Based on 200 answered questionnaires and several Delphi rounds, the investigators concluded bradykinesia/hypokinesia, tremor, sway, gait, sleep, and cognition to be the most important symptoms to assess continuously. Recently another study reported on a detailed patient engagement exercise to develop a set of PRO-items, for both motor and cognitive domains, specifically tailored for early-disease-stage PD patients (Morel et al., 2023). The validation of these items is currently under investigation in follow-up research. The findings of these two studies using patient engagement did not majorly differ, which underlines the generalization of their results (Ferreira et al., 2015; Morel et al., 2023). This, however, does not mean that smaller patient engagement methods are not necessary anymore for specific PD populations. In general, investigators should repeat and check generalized assumptions around data-driven monitoring or prediction models when applying it on new (sub) populations (Kelly et al., 2019), as we will highlight later in this literature overview.

We will provide a brief overview of the current advances to monitor these symptoms both subjectively and objectively. We limit ourselves to methods that are specifically designed, or proven to be feasible, for naturalistic monitoring with a high temporal resolution.

*Subjective* assessments typically consist of PROs and patients should complete them on various temporal intervals, depending on the applied methodology. The best-known diary method is probably the Hauser diary, that differentiates between off- and on-dopaminergic states, with and without (burdensome) dyskinesia (Hauser et al., 2006). Although this method is successfully implemented for repetitive motor symptom assessment throughout a day (Rodriguez-Molinero et al., 2018), it does not provide specific information about all symptomatic items. To collect several specific symptom and functionality domains multiple times daily, ecological momentary assessments (EMA) are introduced and proven to be feasible for Parkinson monitoring (Heijmans et al., 2019; Habets et al., 2020). A second, independent group recently reproduced the feasibility of EMA for PD patients (Weizenbaum et al., 2022). Recent studies including patient experts revisited PRO-items resulting in general advises and two updated PRO instruments (Morel et al., 2023). Since these repetitive PRO methods have different sampling frequencies than the gold standard assessments, naturalistic validation is challenging. A recent study shows, however, that subjective PROs do correlate with traditional gold standard on a single momentary assessment (von Below et al., 2023). Furthermore, structured and explicit instructions proven to increase the correlation between PRO-items and clinical assessments, also for symptoms that are notoriously hard to self-assess, such as dyskinesia (Timpka et al., 2022; Janz et al., 2023).

*Objective* assessments of the listed symptoms have been developed and tested for many years, but the validation of continuous measures throughout a day against a gold standard assessment is often limited (Sica et al., 2021; Vanmechelen et al., 2022). Some IMU-based proprietary methods reported good correlations of passively generated symptom assessments and traditional gold standard assessments on larger temporal intervals (Guan et al., 2021; Rodríguez-Martín et al., 2022). However, some passive assessments correlate better with gold standards on larger rather than shorter temporal intervals (Joshi et al., 2019), where others only report their performance over larger periods of time (Powers et al., 2021). These limitations challenge the interpretation of these assessments over short time intervals. An increasing number of studies reports on the application of active, sometimes gamified, motor assessments either to assess naturalistic motor or cognitive symptoms (Adams et al., 2023; Broeder et al., 2023; Crook-Rumsey et al., 2023; Liikkanen et al., 2023), or to improve symptoms due to training exercises (Gallou-Guyot et al., 2022). They mostly report good feasibilities, some requiring remote support, good test accuracies (Broeder et al., 2023), but also remaining challenges around the interpretation (Page et al., 2022; Liikkanen et al., 2023).

Although the perception and interpretation of cardinal PD symptoms may be generalized largely over PD population globally, it is advised to test the validity of assumed relevant variables in local, independent populations before applying them in predictive modeling (Kelly et al., 2019).

Sauerbier et al. (2017) discussed a large heterogeneity in self-reporting of motor and non-motor symptoms in PD, but that mostly, reliable information on non-white patients is lacking. The FIRE-PD study supported engagement of underrepresented groups (Sanchez et al., 2022), with results not yet published. A very recent study investigated the perception of dyskinesia in different cultures and even stated possible language-based influences on self-reports vs. clinical examination (Kaasinen et al., 2023).

This underlines the importance of reassuring a specific geographical, socio-economic, or cultural PD population agrees with the validity of monitoring methods merely based on existing literature.

It is particularly important to evaluate the inclusivity of the monitoring methods for underrepresented groups among the PD population, since large studies often do not reflect the preferences of these groups (Sanchez et al., 2022).

## Patient engagement in DBS: concepts and preliminary outcomes

### Objectives of patient engagement

We established two primary goals for our patient engagement initiatives:

**Enhanced insight:** Our intention is to gain a clearer understanding of patient preferences and the potential burdens they face, whether from active participation or passive naturalistic data collection.

**Comprehensive experience capture:** We aim to ensure that no vital patient experiences go unnoticed. Our multifaceted monitoring methods should wholly represent patients’ symptoms and overall functionality.

Accomplishing these objectives is anticipated to boost study feasibility and participant adherence, leading to superior data integrity. Here, we will now report on the methodology and the resulting consequences of patient engagement applied, briefly introducing our study design before the patient engagement as the basis for this use case.

#### Study design

We planned to investigate electrophysiological biomarkers in PD patients treated with bilateral subthalamic DBS in a chronic setting. To better interpret the naturalistic neural biomarkers, we ask the participants to use naturalistic monitoring methods in their real-life situation, resulting in objective and subjective measures of symptom severity and general context parallel to the neural recordings. This multi-modal, high intensity data acquisition will be applied during blocks of 2 to 4 weeks, and will be complemented with clinically validated motor and non-motor assessments during hospital visits.

For the subjective assessments, we composed an EMA questionnaire with 16 items covering motor-, non-motor-symptoms and contextual information, based on the available literature (Habets et al., 2020; Morel et al., 2023). A custom-made smartphone-application will prompt the questionnaire on six semi-random times throughout the day (VirgoBit UG, Muenster, Germany).

For the objective assessments, we provide the patients with a commercially available wearable wrist sensor (CardioWatch, Corsano B.V., The Hague, Netherlands) that records heart rate derived measures, activity proxies, sleep staging, and raw accelerometer data. To collect neurophysiological recordings, we will use a passive recording setting from a sensing-enabled internal pulse generator that records one mean value in a pre-defined frequency-bin every 10 min. Besides this passive recording, we will ask the patients to perform an active neurophysiological recording at every EMA-completion.

#### Methodology of patient engagement

The developed patient engagement concept covers active patient involvement throughout the whole study duration (see Figure 1B). First, we will consult a small group of patients during the conceptualization and design of the study. Based on availability and travel distances, we will ask some of these patients to pilot the proposed recording protocol for 2 weeks. After the piloting phase, we will again consult these patients in a group event, to collect feedback and discuss our learned lessons. During the actual study, we will continue to include a group of patients through a “patient expert advisory board.” We will consult and inform this advisory body regarding practical issues and scientific progress via repetitive events. Here, we will discuss our design, preparation, and results from the initial patient engagement meeting, as well as first experiences from the second patient engagement meeting.

We invited PD patients treated with subthalamic DBS that would meet all inclusion criteria for our upcoming study. To maintain a familiar and approachable atmosphere, we made sure the group size did not exceed ten patients. We ensured to have a diverse patient subset regarding age, gender, and general technology affinity. To reduce the patient burden, we included patients with travel times less than an hour. We invited patients to bring an accompanying person of choice. Patients often brought a partner, relative, child, parent, or close friend. Finally, nine patients accepted our invitation.

The goal of the initiating meeting was to discuss feasibility of the planned study and assess unmet needs or concerns of the participants.

The agenda started with a brief update on the current state of research relevant to the planned study to bring everyone to a similar basis for discussion. Therefore, we explained current findings and challenges of biomarker research and introduced the planned selected methods in the prospective study.

After this, we had four breakout sessions with two groups, discussing with scientists and clinicians. We pre-selected four discussion topics that demanded patient and caregiver input:

• Passive and active sensor-based data collection
  ○ *Would wearing a wearable continuously be feasible?*
  ○ *Which device specifications would be important for you (recorded measures, waterproofness, recharging)?*
  ○ *Would self-induction of recordings with an additional device be tolerable?*
  ○ *Would you participate in additional measures such as a video game and what should that look like?*
  ○ *What would be your preference for data transfer?*
• Ecological momentary assessment
  ○ *Are the questions understandable, do you have doubts regarding interpretations?*
  ○ *Do you miss symptoms or functionality assessed?*
  ○ *Do you think the number of items, the frequency or questionnaires, and the EMA method are feasible?*
• Study design and support
  ○ *Would you like a patient advisory board and what should its role be?*
  ○ *Do you prefer study set up meetings at home or in the hospital; would you like to bring a supporting person?*
  ○ *How do you prefer to communicate in case of questions during participation?*
  ○ *What do you think of the current user interface and the feedback it is providing?*
• Open points and further ideas
  ○ *Are any points/symptoms that are important for you missing?*
  ○ *What are your thoughts on further smartphone-based measures?*
  ○ *In what form would you like to receive research results?*
  ○ *How did you like today’s meeting and would you like scientific meetings in future?*

#### Results and consequences of patient engagement meeting

The overall results of feedback concerning the different points are summarized in Table 1.

**TABLE 1:** Patient feedback on breakout-session topics.

Passive/active data collection	EMA
• As little additional devices as possible → 8/9 patients owned smartphones, 8/8 would use their own device for monitoring • Afraid to lose data, as passive data collection as possible is preferred • Active collection should be as gamified as possible, opinions on frequency vary between several times per day to weeks, event recording may be too much	• 2 weeks, multiple times per day may be feasible, if there is no pressure for continuous and complete documentation • Formulation of questions need clarification whether applying to PD-specific symptoms or general well-being vs. general. Supported question-instructions are required at study start. • Proposed user interface was okay, but adjustable font size would be desirable • Visual feedback: beyond a track record of EMA completion, biometric data like steps or heart rate would be desirable
**Study support**	**Additional points**
• Proactive calling/visiting is desired by most • Personal setup visit (including caregiver), good manuals for at home • Study email address and phone for contact • Patient expert panel appreciated as a “backup trust person,” but a dedicated infrastructure including medical professional support would be preferred	• As little personal data as possible Global Positioning System (GPS, app usage) • Very critical about passive measures, e.g., typing speed, in this case, active engagement with e.g., games would be preferred • Regular information events/accessibility of scientific results desirable

Regarding sensors, the main concern of participants was a user friendliness in the sense that it should not need recharging too frequently and should be robust enough to be worn continuously (e.g., also during showering) to avoid data loss due to damage of the sensors or forgetting to reaffix it.

Active data acquisition should be kept as simple as possible—additional devices were seen critically, but a small motor task as a game was received positively. Here, the unanimous opinion was that it should be as gamified as possible and that features like a high score would increase motivation.

When asked whether passive measures, such as typing speed, could in their opinion reliably be used instead, they preferred active measures.

Initially, patients were skeptical about the high intensity recording phases. However, they showed more confidence in these methods after highlighting the scientific value of monitoring data with high temporal resolutions for shorter periods of time (e.g., weeks) compared to only daily measures for longer periods (e.g., months).

The patients preferred very explicitly formulated EMA-items that clarified whether items focus on PD symptoms or on the general wellbeing. The use of solely Likert-scalers and multiple-choice questions was positively accepted.

Interestingly, many patients and relatives had concerns regarding personal data and data transfer. It became clear that for the matter of data security, but also for concerns of data loss, a local saving or automatic transfer to a secure local server would be preferable.

There seems to be a large demand for study support. This implies both a clinical team for medical backup, but also patient experts for representation of the patient perspective and “everyday aspects” of the data acquisition. Since there was an individual variability of preferences, it was advised to offer the study support via study phone and email. Also, there was a strong demand for being involved in the whole scientific process: scientific meetings, updating on the study progress, results and interpretation of data at regular intervals was desired.

The insights from our patient engagement session led to significant modifications in our study design. Practically speaking, even though the original application was well-received, we optimized its framework. The final version now consists of 13 questions: 1 general, 5 on motor symptoms/medication, 3 addressing non-motor symptoms, and 4 contextual inquiries. We also enhanced the application’s home screen based on user feedback, displaying the previous week’s participation scores, cumulative step count, and average heart rate. Additionally, we’ve incorporated battery life details for the wearable sensor and a recharging notification. We agreed with the patients on trying out active measures, e.g., initiation of electrophysiological recordings during the piloting phase, to test the theoretical concerns in practice.

Patients responded positively to study support via patient manuals and several communication ways (phone, email). There will be an individual setup meeting with each participant and at least one relative/caregiver. During this, we will explain the study components with different devices, questionnaires and our support structure. With structured control questions, we will assess whether patients understood the key information. Addressing feedback on individual preferences on location of the setup meeting, we give patients the possibility of an in-clinic or an at-home visit. We also agreed on a second patient engagement meeting after the piloting phase, during which feedback is assessed and a final study protocol is agreed upon (see Figure 1B). There, also the first results will be discussed, as will be the case in update meetings twice per year during the main study.

After a piloting phase of on average 5–7 days with five patients, we had another patient engagement consultation. Here, we received feedback regarding the feasibility of the study design. The overall design with active and passive measures was positively received, and a duration of two-weeks home monitoring was favored. The concern that these times should be planned according to participants’ schedules was raised, e.g., that times with vacation should be avoided in order to capture the “everyday life.” The questionnaires were experienced as not disturbing, with average completion times around < 120 s. Regarding the wearable, the most important consideration was to ensure continuous data acquisition with as little technical demands to the patients as possible. For some patients, too many parallel technical devices (e.g., if not the personal phone could be used) were considered stressful. We agreed on a procedure with the study protocol, including another brief piloting phase.

## Concluding remarks

We here would like to underline the importance and scientific potential of patient engagement methods to enhance naturalistic neuromodulation research in general. More specifically, our patient engagement use case in a naturalistic monitoring study for PD DBS can help researchers to close the gap between the real-life data they desire, and the feasible data and real-life circumstances in which patients collect these data. The reported experiences while preparing, executing, and evaluating our patient engagement activity may guide colleague researchers in movement disorders and/or DBS communities that consider including patient engagement in their studies.

Due to the novelty of multi-modal naturalistic monitoring with high-resolution data collection, it was very valuable to hear patients’ general perceptions of our study design in a different setting than an informed consent conversation. Our impression was that the attending patients felt free to give their honest opinion, since they were considered the “experts” throughout this whole activity.

After our patient engagement session, we revised multiple elements of our study. We pinpointed the key symptoms patients deemed vital to track. While we made subtle tweaks to the monitoring app’s user interface, like font size adjustments and biomarker feedback, a standout insight was the emphasized need for a study support structure. We incorporated this, firmly believing it will be pivotal to bolster patient compliance. We generally found that the patient engagement meeting was also valuable for patient empowerment to foster study motivation and tailor a study design to individual needs. For example, the willingness for high resolution active input was increased when participants understood the scientific necessity, and also realistic burden of e.g., completing the questionnaires. With the second patient engagement meeting, we yielded valuable improvements of our study protocol for long-term feasibility of high-quality chronic data acquisition in the main study. Hence, we recommend for chronic studies to implement a multi-layered diverse patient engagement and support structure with individual and group feedback and implementation meetings.

## Data availability statement

The raw data supporting the conclusions of this article will be made available by the authors, without undue reservation.

## Ethics statement

The studies involving humans were approved by the Ethics Committee of the Charité – Universitätsmedizin Berlin (EA2/256/60). The studies were conducted in accordance with the local legislation and institutional requirements. The participants provided their written informed consent to participate in this study.

## Author contributions

LF: Conceptualization, Data curation, Funding acquisition, Investigation, Methodology, Project administration, Visualization, Writing – original draft, Writing – review & editing. JR: Conceptualization, Supervision, Writing – review & editing. AK: Resources, Supervision, Writing – review & editing. JH: Conceptualization, Data curation, Funding acquisition, Investigation, Methodology, Project administration, Writing – original draft, Writing – review & editing.
